# Expression of an Epitope-Tagged Virulence Protein in *Rickettsia parkeri* Using Transposon Insertion

**DOI:** 10.1371/journal.pone.0037310

**Published:** 2012-05-18

**Authors:** Matthew D. Welch, Shawna C. O. Reed, Rebecca L. Lamason, Alisa W. Serio

**Affiliations:** 1 Department of Molecular & Cell Biology, University of California, Berkeley, California, United States of America; 2 Microbiology Graduate Group, University of California, Berkeley, California, United States of America; University of Illinois at Chicago College of Medicine, United States of America

## Abstract

Despite recent advances in our ability to genetically manipulate *Rickettsia*, little has been done to employ genetic tools to study the expression and localization of *Rickettsia* virulence proteins. Using a *mariner*-based *Himar1* transposition system, we expressed an epitope-tagged variant of the actin polymerizing protein RickA under the control of its native promoter in *Rickettsia parkeri*, allowing the detection of RickA using commercially-available antibodies. Native RickA and epitope-tagged RickA exhibited similar levels of expression and were specifically localized to bacteria. To further facilitate protein expression in *Rickettsia*, we also developed a plasmid for *Rickettsia* insertion and expression (pRIE), containing a variant *Himar1* transposon with enhanced flexibility for gene insertion, and used it to generate *R. parkeri* strains expressing diverse fluorescent proteins. Expression of epitope-tagged proteins in *Rickettsia* will expand our ability to assess the regulation and function of important virulence factors.

## Introduction

*Rickettsia* are Gram-negative alphaproteobacteria that have an obligate intracellular growth requirement and infect arthropod vectors and mammalian hosts. *Rickettsia* species are classified into four groups – the ancestral group (AG), typhus group (TG), transitional group (TRG), and spotted fever group (SFG) [1], [2]. The SFG consists of organisms that cause spotted fever illness, including *Rickettsia parkeri*, a species that was originally considered to be nonpathogenic, but is now recognized as a causative agent of mild or moderate disease in North and South America [3], [4].

Despite clinical and biological interest in *Rickettsia*, tools for their genetic manipulation have only recently been implemented. In particular, it is now possible to transform *Rickettsia* with DNA [5]–[7], introduce transposons into their genomes [8], [9], carry out directed mutagenesis [10], and stably transform them with plasmids [11]. These advances have been used to express exogenous proteins, such as fluorescent protein variants [6], [8], [9], [11], [12]. However, little has been done to employ these tools to study the expression, localization and function of endogenous *Rickettsia* proteins.

Because of their obligate intracellular growth niche, examining the expression and localization of *Rickettsia* proteins that interface with the host cell is crucial for understanding pathogenesis. For SFG *Rickettsia* such as *R. parkeri*, both invasion and spread involve an interaction with the host cell actin cytoskeleton. During invasion, actin filament polymerization underlying the host cell plasma membrane promotes bacterial phagocytosis [13], [14]. Following escape from the phagosome, bacteria use the force from actin polymerization on their surface to propel them through the cytosol, a process that results in the formation of actin comet tails [15], [16]. Movement drives bacteria into protrusions of the host cell plasma membrane, leading to escape [17] or internalization by an adjacent cell [18]. The RickA protein has been implicated in actin assembly by *Rickettsia* [19], [20]. RickA mimics the activity of host proteins called nucleation promoting factors (NPFs) by binding to and activating the host Arp2/3 complex, an actin nucleating and organizing factor [19], [20]. The *rickA* gene is present in AG, TRG and SFG species, but is absent in TG *Rickettsia* [21]–[25]. Further elucidation of the role of RickA and other *Rickettsia* proteins in infection will require the development of tools to monitor their expression and localization.

Here, we report the implementation of epitope tagging as a tool for observing protein expression and localization in *Rickettsia*. Using a *mariner*-based *Himar1* transposition system [9] in *R. parkeri*, we expressed a FLAG-tagged variant of RickA under the control of its native promoter, allowing for the detection of RickA using commercially-available antibodies. To further facilitate protein expression in *Rickettsia*, we developed a new plasmid containing a variant *Himar1* transposon with enhanced flexibility for gene insertion, and used it to generate *R. parkeri* strains expressing fluorescent proteins. These tools will facilitate future studies aimed at assessing the function of *Rickettsia* virulence factors.

## Results

### Transformation of *R. parkeri* for fluorescent protein expression

**Figure 1:**
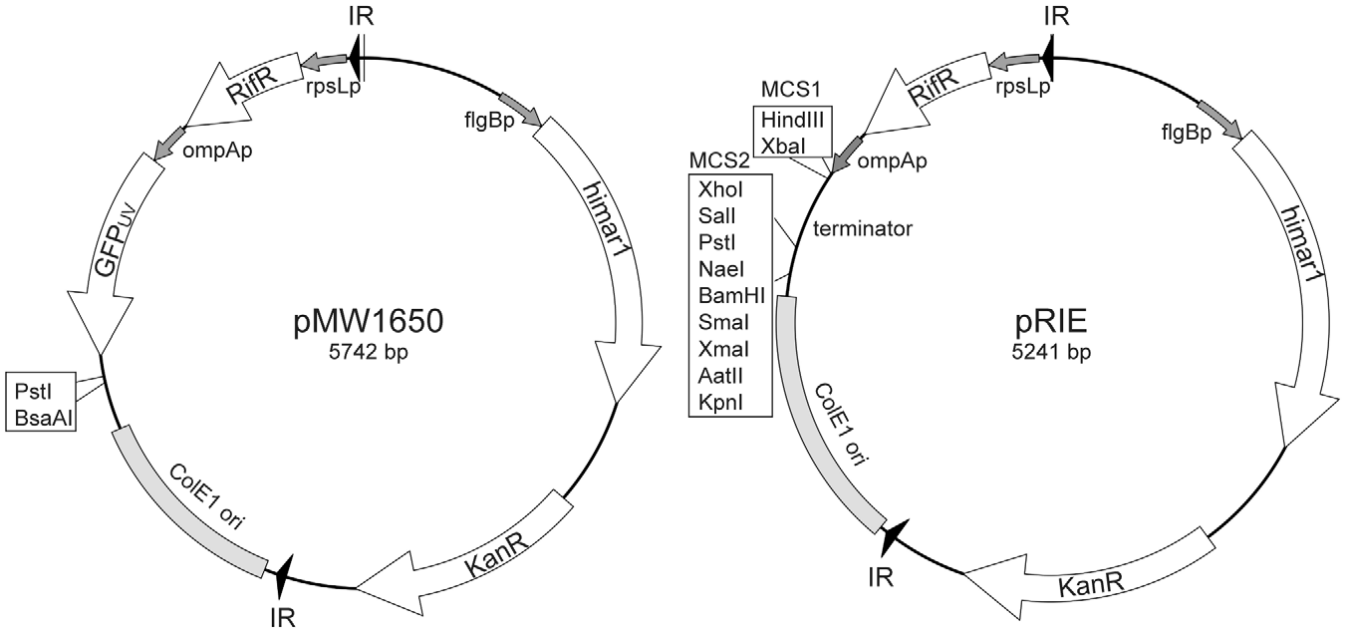
Physical maps for pMW1650 and pRIE. Both pMW1650 [9] and pRIE contain a *Himar1* transposase gene under the control of the *Borrelia burgdorferi flgB* promoter, and a kanamycin resistance gene (KanR). Within the transposable element, bounded by inverted repeats (IR), are an *E. coli* ColE1 origin of replication, a GFP_UV_ gene under the control of the *R. rickettsii ompA* promoter, and a rifampicin resistance gene (RifR) under the control of the *rpsL* promoter. In addition, pRIE contains two multiple cloning sites: MCS1 for insertion of fluorescent protein genes under the control of the *R. rickettsii ompA* promoter, and MCS2 for insertion of other genes to be expressed under the control of their native promoter.

We sought to transform *R. parkeri* to express recombinant proteins, including variants of both green and red fluorescent protein, as well as epitope-tagged *Rickettsia* proteins. For transformation we first employed a previously-developed *mariner*-based *Himar1* transposition system that is contained on the plasmid pMW1650 (Figure 1) [9], which is engineered to express the green fluorescent protein variant GFP_UV_ [26]. To demonstrate the feasibility of transforming *R. parkeri*, we electroporated bacteria with pMW1650 and selected for rifampicin-resistance. Antibiotic-resistant strains were isolated, examined for expression of GFP_UV_ by fluorescence microscopy, and plaque purified. Strains were further characterized by sequencing the site of transposon insertion and locating the sequence in the *R. parkeri* genome (GenBank accession number CP003341). Two independent strains were isolated (Table 1), each with the transposon inserted at a different genomic location.

**Table 1:** Transposon insertion sites for *R. parkeri* strains.

Strain	Insertion site	Insertion site gene
pMW1650-1	752,507–752,510	between MC1_40245 and MC1_40250
pMW1650-2	14,101–14,104	between MC1_t07582 and MC1_00100
pMW1650-FLAG-RickA-1	663,623–663,626	In MC1_03780
pMW1650-FLAG-RickA-2	612,772–612,775	In MC1_03530
pMW1650-FLAG-RickA-3	772,629–772,632	In MC1_04385
pRIE-EGFP-1	210,826–210,827	In MC1_01110
pRIE-mCherry-1	220,664–220,700	In MC1_01155
pRIE-3XmCherry-1	1,203,629–1,203,630	In MC1_07005
pRIE-3XmCherry-2	1,237,065–1,237,066	In MC1_07155

To facilitate the generation of *R. parkeri* strains that express different fluorescent proteins, as well as recombinant versions of endogenous *Rickettsia* proteins, we developed a new variant of plasmid pMW1650 called plasmid for *Rickettsia* insertion and expression (pRIE) (Figure 1). In addition to the features in pMW1650, pRIE contains two multiple cloning sites: MCS1 for strong expression of genes, such as those encoding fluorescent proteins, under the control of the *R. rickettsii ompA* promoter; and MCS2 for expression of a *Rickettsia* gene under the control of its native promoter or another promoter of choice. To test the utility of pRIE, we generated versions containing genes encoding enhanced GFP [27] (pRIE-EGFP), the red fluorescent protein variant mCherry [28] (pRIE-mCherry), or three tandem copies of mCherry (pRIE-3XmCherry). *R. parkeri* were electroporated with each pRIE variant, antibiotic-resistant strains expressing the fluorescent proteins were plaque purified, and the site of transposon insertion was determined. For each, at least one independent strain was isolated (Table 1).

**Figure 2:**
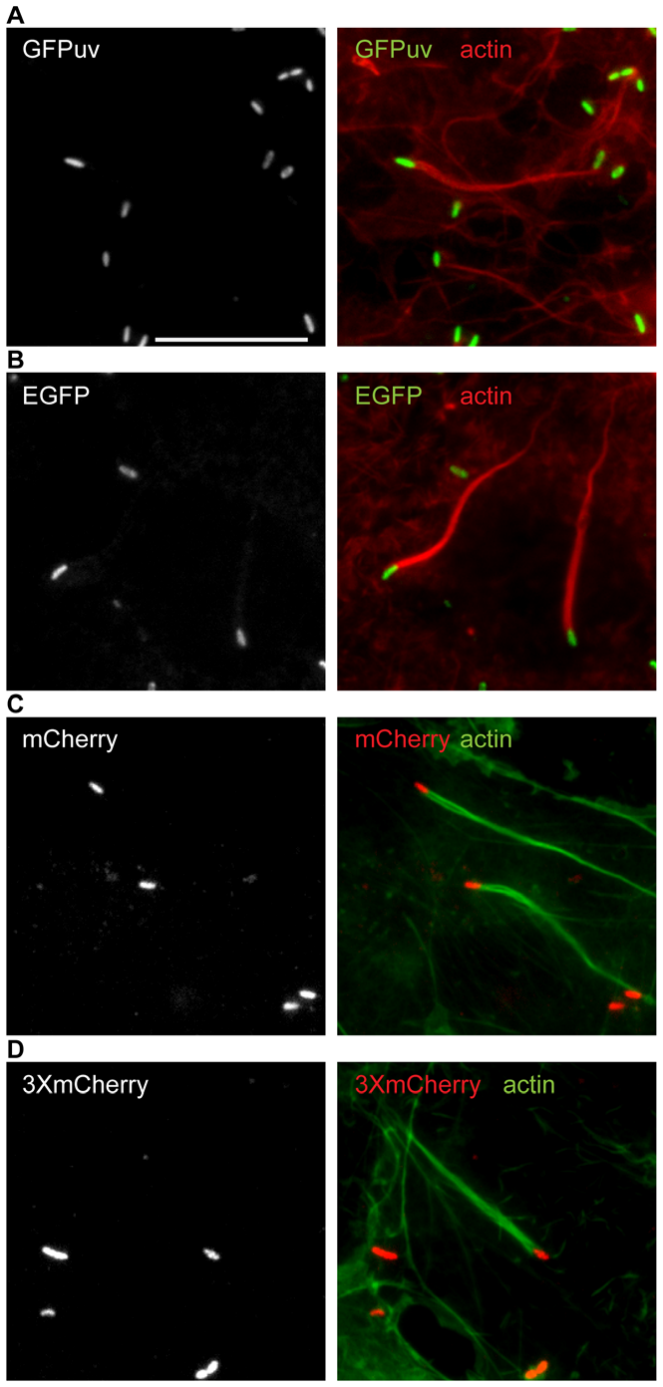
Expression of fluorescent proteins in *R. parkeri*. *R. parkeri* strains expressing (A) GFP_UV_, (B) EGFP, (C) mCherry, and (D) 3XmCherry are shown in infected Cos7 cells alone (left) and together with actin filaments (right). Colors are indicated in the image. Scale bar 10 µm.

When cells were infected with representative strains transformed with either pMW1650 or pRIE, expression of fluorescent proteins was readily observed (Figure 2). Thus, the pRIE transposon system is useful for expressing diverse fluorescent proteins either singly or as tandem fusions. Notably, all strains formed actin comet tails in cells (Figure 2), suggesting that transposon insertion and fluorescent protein expression does not affect the process of actin-based motility.

### FLAG-RickA expression in *R. parkeri*

We next sought to express a recombinant, epitope-tagged version of the endogenous *R. parkeri* RickA protein, which would enable protein detection and isolation using commercially-available antibodies. The epitope tag we chose was the FLAG peptide (DYKDDDDK), which is recognized by antibodies useful for immunoblotting, immunofluorescence microscopy, and protein purification [29]. The gene encoding FLAG-RickA under the control of the native RickA promoter was subcloned into the PstI and BsaAI sites in pMW1650, between the GFP gene and the ColE1 origin of replication (Figure 1), generating pMW1650-FLAG-RickA. The plasmid was then introduced into bacteria by electroporation, followed by antibiotic selection, visual screening for GFP_UV_ expression, and plaque purification. Three independent strains were isolated, each with the transposon in a different genomic location (Table 1).

**Figure 3:**
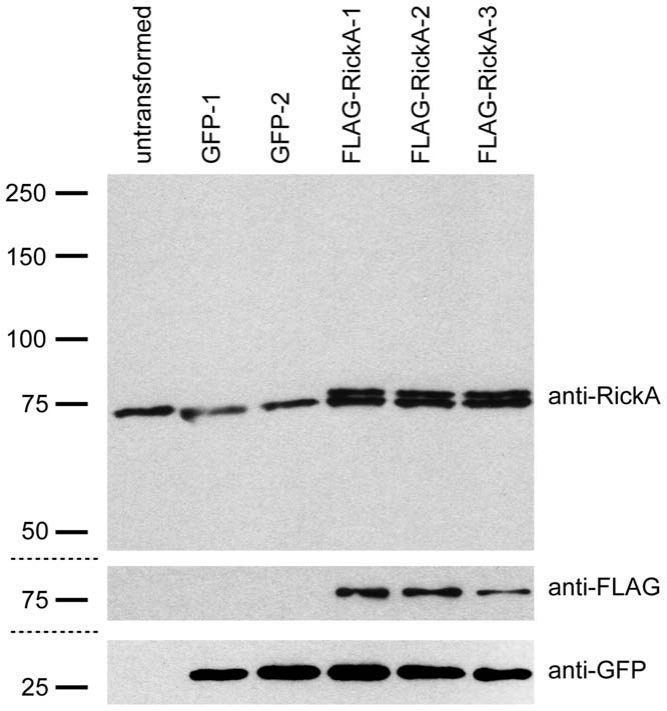
Expression of FLAG-RickA in *R. parkeri*. Immunoblots of *R. parkeri* strains that are not transformed (untransformed), transformed with pMW1650 to express GFP_UV_ (GFP-1, 2), and transformed with pMW1650-FLAG-RickA to express GFP_UV_ and FLAG-RickA (FLAG-RickA-1, 2, 3), probed with anti-RickA (top blot; higher molecular mass band is FLAG-RickA, lower is endogenous RickA), anti-FLAG (middle blot) or anti-GFP antibodies (bottom blot). Equal volumes were loaded into each lane, and the levels of endogenous RickA serve as a built in loading control.

The expression of FLAG-RickA, endogenous RickA, and GFP, was assessed in untransformed bacteria, in strains transformed with the parent plasmid pMW1650, or strains transformed with pMW1650-FLAG-RickA (Figure 3). All strains expressed endogenous RickA at similar levels, as assessed by immunoblotting using anti-RickA antibodies [20]. All transformed strains expressed GFP_UV_ at similar levels. Strains transformed with pMW1650-FLAG-RickA also expressed FLAG-RickA, as detected by immunoblotting with anti-FLAG antibody. FLAG-RickA could be separated from native RickA by SDS-PAGE because of its slightly larger molecular mass, and was expressed at levels similar to those of native RickA as detected using anti-RickA antibodies. Thus, epitope-tagged *Rickettsia* proteins can be expressed from their native promoters at endogenous levels, and can be readily detected by immunoblotting using commercially-available antibodies.

**Figure 4:**
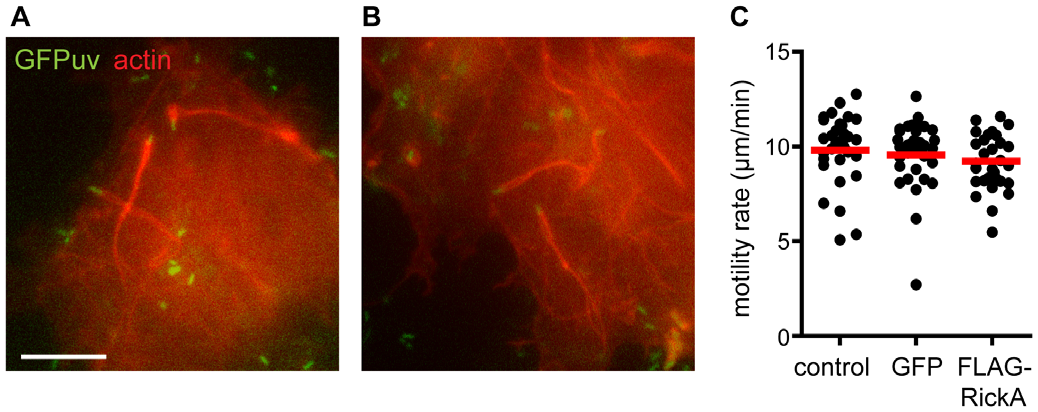
Expression of FLAG-RickA does not affect actin-based motility. *R. parkeri* strains (green) transformed with (A) pMW1650 to express GFP_UV_, or (B) pMW1650-FLAG-RickA to express GFP_UV_ and FLAG-RickA, were imaged in live Cos7 cells expressing the actin marker mCherry-Lifeact (red). Scale bar 10 µm. (C) Scatter plots of the rates of actin-based motility for untransformed bacteria (control) or strains expressing GFP_UV_ or GFP_UV_ and FLAG-RickA.

Given that RickA has been proposed to function in actin-based motility [19], [20], we examined whether expression of FLAG-RickA affects this process. The association of bacteria with actin and the rates of movement were compared for representative strains transformed with pMW1650 (GFP-2) or pMW1650-FLAG-RickA (FLAG-RickA-2) (Figure 4). Both strains formed actin comet tails and moved at mean rates that were statistically indistinguishable from untransformed control bacteria (motility rates mean +/− standard deviation: control, 9.8+/−1.8 µm/min; GFP-2, 9.7+/−1.7 µm/min; FLAG-RickA-2, 9.2+/−1.5 µm/min). Thus the expression of FLAG-RickA did not affect actin-based motility.

**Figure 5:**
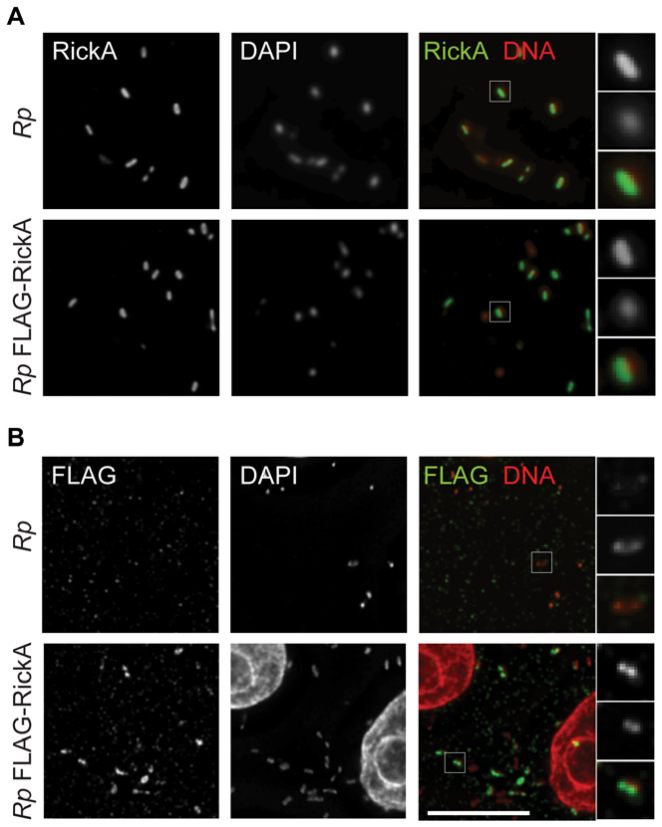
Detection of FLAG-RickA in bacteria. *R. parkeri* strains that were not transformed (*Rp*) or transformed with pMW1650-FLAG-RickA (*Rp* FLAG-RickA) were used to infect Vero cells and then (A) labeled by immunofluorescence with anti-RickA antibody and stained for DNA with DAPI, or (B) labeled by immunofluorescence with anti-FLAG antibody and stained for DNA with DAPI. In the merged images, RickA or FLAG are labeled in green, and DNA in red. Scale bar 10 µm. Higher magnification images of individual bacteria (highlighted in boxes in the lower magnification images) are shown on the right.

To assess whether FLAG-RickA can serve as a marker for RickA in *R. parkeri* cells, we assessed the presence of RickA by immunofluorescence microscopy (Figure 5). RickA could be detected associated with the majority of *R. parkeri* using anti-RickA antibodies. Similarly, FLAG-RickA was detected in FLAG-RickA-expressing bacteria using anti-FLAG antibody, but was not detected in untransformed bacteria. RickA and FLAG-RickA were detected in most but not all bacteria, suggesting that there are differences in RickA expression or antibody accessibility in individual bacteria within the population. These results indicate that FLAG-RickA can be used as a marker for endogenous RickA in bacterial cells.

## Discussion

Our results indicate that epitope-tagged proteins such as FLAG-RickA can be expressed at similar levels to their endogenous counterparts in *Rickettsia* and can thus be used as markers for protein expression and localization. RickA has been implicated in actin-based motility [19], [20], [30] and suggested to be a factor that might promote actin assembly during invasion of host cells [31]. In previous studies, RickA has been reported to be localized on the bacterial surface by immunofluorescence microscopy [19], [32], and in association with the inner and outer membranes as well as in the extracellular space by immunoelectron microscopy [32]. In our experiments, both FLAG-RickA and endogenous RickA were detected in association with bacteria in a pattern consistent with these previous findings. Interestingly, we detected FLAG-RickA and RickA associated with most but not all bacteria, suggesting that RickA expression may vary during *Rickettsia* infection or the bacterial cell cycle. The use of epitope-tagged variants of RickA should facilitate future studies aimed at further examining its expression, biochemical interactions, localization and function during infection.

To facilitate the expression of epitope-tagged proteins in *Rickettsia*, we developed an improved variant of plasmid pMW1650 [9], called pRIE, which harbors a *Himar1* transposon with enhanced flexibility for gene insertion. The transposon in pRIE contains two multiple cloning sites, one for insertion of genes encoding a fluorescent protein of choice under the control of the *R. rickettsii ompA* promoter, and the other for insertion of any gene encoding an epitope-tagged or untagged protein under the control of its native promoter or another promoter of choice. Because the transposon integrates into the genome in a single copy, it offers the advantage of expressing an epitope-tagged protein at similar levels to its endogenous counterpart. This contrasts with recently developed *Rickettsia* shuttle plasmids, which are present in higher copy number in transformed bacteria [11], and are thus likely to overexpress proteins. A potential disadvantage of the transposon-based system, however, is that introducing transposons into the *Rickettsia* genome often disrupts genes, and in our experiments all but one of the insertion sites were in predicted coding sequences. Nevertheless, we did not observe any phenotypic consequences of transposon insertion on bacterial growth or actin-based motility. Moreover, transposon insertion has been used to complement mutations in *R. rickettsii* [33], suggesting that genes expressed from transposons can maintain their normal regulation and function. Thus, transposon insertion is a viable method for expressing proteins in *Rickettsia*, and the design of pRIE will facilitate expression of multiple genes from separate promoters for protein localization and genetic complementation experiments.

Transformation of *Rickettsia* with transposons that express epitope-tagged and fluorescent proteins can be added to a growing list of genetic manipulations that have recently been implemented, which also include directed mutagenesis [10] and stable transformation with plasmids [11]. Taken together, these advances will expand our ability to define the factors important for *Rickettsia* virulence and characterize the mechanisms through which they impact the host cell.

## Materials and Methods

### Plasmids

pMW1650 (Figure 1) was a gift from D. Wood (University of South Alabama, Mobile). To generate pMW1650-FLAG-RickA, the upstream 300 bp of genomic sequence was amplified from boiled *R. parkeri* by PCR using primers 5′-CCGCACGTACATCCGTAATTTTACAGGTATATTAC-3′ and 5′-CCTTTATCATCATCATCTTTATAATCCATGCCATATACCTACTATAAATTAATATCAG-3′, which included a sequence encoding part of the FLAG tag. The coding sequence for RickA was amplified using primers 5′- GGATTATAAAGATGATGATGATAAAGGTACCGCGGGTATGGTTAAAGAAATAGATATAAATAAATTATTAG-3′ and 5′-CATTTAACTAGTTTATCTAACAAATGATGGTTTTTGTG-3′, which also included a sequence encoding part of the FLAG tag. These PCR products were combined and a fusion gene was generated by PCR using primers 5′-CCGCACGTACATCCGTAATTTTACAGGTATATTAC-3′ and 5′-CGCCTGCAGTAATTTCTTTGGTAAAAGAATAGTTGAAATAACTAAGAAGTTTAAAGGGTAGATTTATATGGTGTTTTATGTCATTCCCACTTCCGTGGGAATGACATTTAACTAGTTTATCTAACAAATGATGGTTTTTG-3′, which also incorporate the RickA 3′ untranslated region. The resulting PCR product was subcloned into the BsaAI and PstI sites in pMW1650.

pRIE (Figure 1) and *Rickettsia* codon-optimized mCherry were synthesized by Genscript. The sequence of pRIE is available in GenBank (accession number JQ638951). *Rickettsia* codon-optimized EGFP was synthesized by Integrated DNA Technologies. To generate pRIE-mCherry and pRIE-EGFP, the mCherry and EGFP genes were subcloned into the HindIII and XbaI sites in pRIE. To generate pRIE-3XmCherry, the mCherry gene was amplified by PCR to generate intervening linkers containing XhoI (between copies 1 and 2) or BglII sites (between copies 2 and 3), and then ligated into pRIE using HindIII and XbaI. All plasmid sequences were confirmed by DNA sequencing at the University of California, Berkeley, sequencing facility.

### Bacterial strains, growth and purification

The *R. parkeri* Portsmouth strain was a gift from C. Paddock (Centers for Disease Control and Prevention, Atlanta). *R. parkeri* was propagated in Vero African green monkey epithelial cells (from ATCC, via the University of California, Berkeley, tissue culture facility) grown at 33°C with 5% CO_2_, and purified by Renografin density gradient centrifugation as described previously [34]. *R. parkeri* clonal strains expressing GFP_UV_, GFP_UV_ and FLAG-RickA, EGFP, mCherry, or 3XmCherry were generated by electroporating freshly-prepared bacteria suspended in 100 µl 250 mM sucrose with 20–25 µg of plasmid DNA (pMW1650, pMW1650-FLAG-RickA, pRIE-EGFP, pRIE-mCherry, or pRIE-3XmCherry, respectively) at 2.5 kV, 200 ohms, 25 µF, for 5 ms using a Gene Pulser Xcell (Bio-Rad). Bacterial viability decreased an average of 4-fold following electroporation, as detected by counting plaque-forming units before and after electroporation. Electroporated bacteria were resuspended in 500–1000 µl brain heart infusion (BHI) media (Difco), and used to infect Vero cells to perform polyclonal purification or direct plaque purification.

For polyclonal purification, 500 µl of electroporated bacteria were used to infect 25–75 cm^2^ flasks of Vero cells. Cells were grown in Dulbecco's modified Eagle's medium (DMEM) with 2% fetal bovine serum (FBS) overnight at 33°C with 5% CO_2_, and after 18–24 h media was replaced with media containing 200 ng/ml rifampicin (Sigma-Aldrich). After 5–7 d, plaques were visible in the cell monolayer. The resulting polyclonal stock was collected, amplified and plaque purified as described below to yield a clonal strain. For direct plaque purification, 100 µl electroporated bacteria was used to infect each well of a 6-well plate of Vero cells and immediately overlaid with 3 ml of DMEM with 5% FBS and 0.5% agarose. Approximately 20 h post infection, 2 ml of DMEM with 5% FBS, 500 ng/ml rifampicin and 0.5% agarose was overlaid on each well. After 4–6 d, plaques were isolated and resuspended in 200 µl of BHI before being used to infect a 25 cm^2^ flask of Vero cells. Strains propagated in this way were collected, and plaque purified a second time before mapping of the transposon insertions sites.

The insertion site of each pMW1650-derived transposon cassette was determined as described previously [9]. Briefly, genomic DNA from *R. parkeri* was digested with HindIII, the restriction enzyme was heat-inactivated, and the DNA fragments were self-ligated. *E. coli* were transformed with the resulting plasmids, and selected for resistance to 100 µg/ml rifampicin. The insertion sites were sequenced using transposon-specific primers 5′-CGCCACCTCTGACTTGAGCGTCG-3′ and 5′-CCATATGAAAACACTCCAAAAAAC-3′, and found to be positions of the *R. rickettsii* Sheila Smith strain [35] listed in Table 1. The insertion site of the pRIE-derived transposon cassette was determined using semi-random, nested PCR. The first round of PCR amplified *Rickettsia* DNA using a universal primer 5′-GCTAGCGGCCGCACTAGTCGANNNNNNNNNNCTTCT-3′ (N is any nucleotide) and transposon-specific primers 5′-CACCAATTGCTAAATTAGCTTTAGTTCC-3′ and 5′-GTGAGCTATGAGAAAGCGCCACGC-3′. The second PCR reaction amplified products from the first round PCR products using internal, transposon-specific primers 5′-GCTAGCGGCCGCGGTCCTTGTACTTGTTTATAATTATCATGAG-3′ and 5′-GCTAGCGGCCGCCCTGGTATCTTTATAGTCCTGTCGG-3′ and a primer 5′-GCTAGCGGCCGCACTAGTCGA-3′ that bound to a portion of the universal primer. PCR products were sequenced, and transposons were found to be positions of the *R. rickettsii* Sheila Smith strain [35] listed in Table 1.

### Cell growth, transfection, and bacterial infection

Cos7 African green monkey kidney fibroblast cells (from Regeneron Pharmaceuticals Inc., via the University of California, Berkeley, tissue culture facility) and Vero cells were grown at 37°C with 5% CO_2_ in DMEM (Invitrogen) with 2–10% FBS (JR Scientific). For expression of Lifeact-mCherry [36] or Lifeact-GFP [31] from plasmid pEGFP-N1, 150 ng of plasmid DNA was transiently transfected into cells using Lipofectamine 2000 (Invitrogen), according to the manufacturer's instructions. For infections, cells were seeded onto glass coverslips in 24-well plates, infected with *R. parkeri*, and incubated at 33°C with 5% CO_2_ for 24–48 h.

### Antibodies, immunoblotting and immunofluorescence staining

Antibodies were obtained from the following sources (in parentheses): mouse anti-*Rickettsia* M14-13 and rabbit anti-*Rickettsia* R4668 and I7205 (T. Hackstadt, NIH/NIAID Rocky Mountain Laboratories; [37], [38]); mouse anti-FLAG M2 monoclonal antibody (Sigma-Aldrich); mouse anti-FLAG M5 monoclonal antibody (Sigma-Aldrich); rabbit anti-RickA [20]; rabbit anti-GFP [39]; horseradish peroxidase conjugated anti-mouse and rabbit secondary antibodies for immunoblotting (GE Healthcare); Alexa 568 anti-rabbit and anti-mouse secondary antibodies for immunofluorescence microscopy (Invitrogen Molecular Probes).

For immunoblotting, bacteria were purified from cultures with similar levels of infection and number of bacteria per cell, purified bacteria were lysed in protein gel sample buffer, equal volumes were separated by SDS-PAGE, and proteins were transferred to nitrocellulose. Membranes were blocked with 5% milk in Tris-buffered saline (TBS), probed with primary antibodies (rabbit anti-RickA, mouse M5 anti-FLAG, rabbit anti-GFP), followed by HRP-conjugated secondary antibodies, and visualized with ECL detection reagents (GE Healthcare).

For immunofluorescence microscopy of RickA, Vero cells were fixed with 2.5% formaldehyde in phosphate-buffered saline (PBS) at room temperature for 15 min, permeabilized in PBS with 0.5% Triton X-100 for 5 min, and then with PBS+1 mg/ml lysozyme (Sigma Aldrich) for 5 min. Primary antibodies were added in PBS+2% bovine serum albumin (BSA), then secondary antibodies were added with together with DAPI (Invitrogen Molecular Probes) to stain DNA. For actin staining, Cos7 cells were fixed with 4% paraformaldehyde in PBS at room temperature for 15 min, permeabilized in PBS with 0.5% Triton X-100, and actin filaments were labeled with 4 U/µl Alexa 568- or 488-conjugated phalloidin (Invitrogen Molecular Probes). Coverslips were mounted with Prolong Gold antifade (Invitrogen Molecular Probes) and stored at 4°C.

### Imaging

Images were captured using a Nikon Eclipse Ti equipped with a 60× (1.49 NA) TIRF objective and an Andor Clara Interline CCD camera (Figure 2), an Olympus IX71 microscope equipped with a 100× (1.35 NA) PlanApo objective lens and a Photometrics CoolSNAP HQ camera (Figure 4), or an Applied Precision DeltaVision 4 Spectris microscope equipped with a 60× PlanApo (1.4 NA) and a Photometrics CH350 CCD camera (Figure 5). Standard images (Figures 2 and 4) were captured using MetaMorph software (Molecular Devices), and then cropped and adjusted for brightness/contrast using Adobe Photoshop or ImageJ. Deconvolution images (Figure 5) were captured using SoftWoRx v3.3.6 software (Applied Precision), deconvolved with Huygens Professional v3.1.0p0 software (Scientific Volume Imaging), and processed using Imaris (Bitplane) and Adobe Photoshop.
